# Use of whole-genome sequencing to trace, control and characterize the regional expansion of extended-spectrum β-lactamase producing ST15 *Klebsiella pneumoniae*

**DOI:** 10.1038/srep20840

**Published:** 2016-02-11

**Authors:** Kai Zhou, Mariette Lokate, Ruud H. Deurenberg, Marga Tepper, Jan P. Arends, Erwin G. C. Raangs, Jerome Lo-Ten-Foe, Hajo Grundmann, John W. A. Rossen, Alexander W. Friedrich

**Affiliations:** 1 Department of Medical Microbiology, University of Groningen, University Medical Center Groningen, Groningen, Netherlands; 2 Department of Rehabilitation Medicine, Center for Rehabilitation, University of Groningen, University Medical Center Groningen, Netherlands; 3 State Key Laboratory for Diagnosis and Treatment of Infectious Diseases, Collaborative Innovation Center for Diagnosis and Treatment of Infectious Diseases, the First Affiliated Hospital of Medicine School, Zhejiang University, China.

## Abstract

The study describes the transmission of a CTX-M-15-producing ST15 *Klebsiella pneumoniae* between patients treated in a single center and the subsequent inter-institutional spread by patient referral occurring between May 2012 and September 2013. A suspected epidemiological link between clinical *K. pneumoniae* isolates was supported by patient contact tracing and genomic phylogenetic analysis from May to November 2012. By May 2013, a patient treated in three institutions in two cities was involved in an expanding cluster caused by this high-risk clone (HiRiC) (local expansion, CTX-M-15 producing, and containing hypervirulence factors). A clone-specific multiplex PCR was developed for patient screening by which another patient was identified in September 2013. Genomic phylogenetic analysis including published ST15 genomes revealed a close homology with isolates previously found in the USA. Environmental contamination and lack of consistent patient screening were identified as being responsible for the clone dissemination. The investigation addresses the advantages of whole-genome sequencing in the early detection of HiRiC with a high propensity of nosocomial transmission and prolonged circulation in the regional patient population. Our study suggests the necessity for inter-institutional/regional collaboration for infection/outbreak management of *K. pneumoniae* HiRiCs.

*Klebsiella pneumoniae* has emerged as an important nosocomial pathogen, known as one of the “ESKAPE” pathogens^1^. Especially, the prevalence of multi-drug resistant (MDR) *K. pneumoniae* increased dramatically in recent years. This limits efficient clinical treatment tremendously resulting in undesirable treatment outcomes.

To control the spread of drug resistance, the tracking of antimicrobial resistant microorganisms is crucial and has been proposed in the CDC antimicrobial resistance action plan as one of the four core actions^2^. Use of multilocus sequence typing (MLST) revealed that the population of MDR *K. pneumoniae* is largely oligoclonal, and some epidemic/endemic clones have been identified. For instance, the extended-spectrum **β**-lactamase (ESBL)-producing *K. pneumoniae* (ESBL-KP) mainly belong to sequence type (ST) 11, ST15, ST101, ST147, and ST336^3^,^4^,^5^,^6^,^7^, and a specific lineage (ST258) played a major role in dissemination of *Klebsiella pneumoniae* carbapenemases (KPC) worldwide^8^. With the rapid development of whole-genome sequencing (WGS), the population structure of MDR pathogens can be dissected at a higher resolution level. This challenges conventional hypotheses on clonal descent of *K. pneumoniae*.

To efficiently control and prevent the dissemination of MDR pathogens in hospital settings, the ability of early detection and the tracking of infected or colonized patients are crucial^9^. This is especially a challenge during a prolonged outbreak, as transmissions between patients may be caused by additional reservoirs (e.g. health-care workers and the environment), and/or in case different institutes and/or cities are involved in the outbreak.

In this study, we present our experience in using whole-genome sequencing and further developing an outbreak clone-specific PCR for tracing and controlling a regional and inter-institutional outbreak caused by a ST15 CTX-M-15-KP clone between May 2012 and September 2013 in the northern part of Netherlands.

## Materials and Methods

### Setting

The University Medical Center Groningen (UMCG) is a 1,300-bed tertiary-care medical centre in the northern part of Netherlands. The rehabilitation centre is part of the university hospital but located 6-kilometer away from the main centre. Most patients of the rehabilitation center were transferred from the university hospital. If a complication occurred or if additional treatment was required, they were admitted to or visiting the hospital again. Part of the medical staff worked both in the hospital and the rehabilitation center.

### Patient tracking and contact tracing

At the end of August 2012, an increase of ESBL-producing *K. pneumoniae* (ESBL-KP) was observed at the UMCG and the associated rehabilitation centre. To prevent further spread, stringent infection control measures consisting of strict patient and staff cohorting were introduced at both sites in August 2012. The outbreak was declared under control in September 2012. Extended infection control measures ended in May 2013. Patients on “high risk” wards, defined as those wards having extensive patient exchange with the ones where positive patients stayed, were screened for ESBL-KP once a week. Patients overlapping with positive patients on the ward were screened twice a week. Discharged patients who overlapped with positive patients between May and September 2012 were traced and retrospectively screened at home using a self-sampling kit. The kit contained two swabs used for the throat and rectum sampling. Instructions about how to use the kit were included. All patients were requested to send the kit back as soon as they took the samples. A case was defined as a patient infected or colonized with an ESBL-producing *K. pneumoniae* after 1^st^ May 2012 sharing the same antibiotic resistance pattern as the outbreak clone.

### Screening and isolates collected in this study

Screening of ESBL-producing Enterobacteriaceae (ESBL-E) was performed as described previously^10^. Briefly, patients were screened for ESBL-E carriage using perianal swabs (Eswab, Copan, Italy), and environmental screening was performed in the patient rooms (e.g. beds) and toilets (e.g. toilet chairs) using MW728 POLYWIPE® sponge swabs (Medical wire & equipment, Wiltshire, England). The swab was plated on a blood agar plate after vortexing, and the liquid Amies eluent was inoculated on selective tryptic soy broth with cefotaxime (0.25 mg/L) and vancomycin (8 mg/L) (TSB-VC). After 18–24 hours of incubation (35–37 °C), 10 μl TSB-VC was sub-cultured on both sides of an Extended Beta-Lactamase Screening Agar (EbSA) plate (AlphaOmega, ‘s-Gravenhage, Netherlands). The EbSA plate consists of a split MacConkey agar plate containing ceftazidime (1.0 mg/L) on one side and cefotaxime (1.0 mg/L) on the other side. Both sides contain cloxacillin (400 mg/L) and vancomycin (64 mg/L) for inhibition of AmpC beta-lactamase-producing bacteria and Gram-positive bacteria, respectively. Subsequently the plates were incubated aerobically at 35 to 37 °C for 18 to 24 hours. Species identification was performed for all oxidase negative isolates that grew on either side of the agar by MALDI-TOF (Bruker Daltonik GmbH, Bremen, Germany). In total, 19 *K. pneumoniae* isolates obtained from patients (KP-1 to KP12, KP-45D, KP-33P, KP-86L, and KP-33F) and environment (KP-1E to KP-3E) were included in this study. The details of isolates are listed in Table 1.

### Conventional testing

Phenotypic susceptibility testing was performed using the Vitek II system with card AST N-199 (BioMerieux, Marcy l′Etoile, France) according to the guidelines of the manufacturer. Breakpoints were interpreted according to EUCAST guidelines (bacteria v4.0). MLST was performed according to the protocol described on the *K. pneumoniae* MLST website (www.pasteur.fr/mlst). The sequence type (ST) was assigned by the MLST database (www.pasteur.fr/mlst/Kpneumoniae.html).

### WGS, de novo assembly and annotation

A total of 19 isolates were sequenced. WGS and *de novo* assembly was performed as described previously^11^. Isolate KP-5 was randomly selected for being scaffolded by mate-pair sequencing. The mate-pair DNA library was prepared using the Mate Pair Library Prep Kit v2 (Illumina) according to the manufacturer’s instructions followed by running on the Miseq for generating 100-bp reads. Scaffolding was performed by SSPACE standard version 3.0 with default settings^12^. Further gaps within scaffolds were attempted to be closed using GapFiller with default settings^13^. The genomes were manually curated after performing automatic annotation on the RAST server^14^.

### Identification of antibiotic resistance-related genes and virulence factors

Three types of drug-resistance genes were investigated, including acquired drug-resistance genes, genes of efflux pumps, and genes associated with drug resistance by specific mutations. Virulence factors studied here include adhesins, capsule production, iron uptake systems, nitrogen source utilization and secretion systems^15^,^16^. The acquired antimicrobial resistance genes were identified by uploading assembled genomes to the Resfinder server v2.1 (http://cge.cbs.dtu.dk/services/ResFinder-2.1/). The other genes relating to resistance and virulence were detected by using the mapping unit of CLC Genomics Workbench to map and/or by blasting assembled genomes to a pseudomolecule generated by concatenating a set of target genes. The capsular genotype was determined *in silico* by *wzi* typing^17^. Scaffolds with resistance-related and virulence genes were blasted against GenBank to identify their genetic location.

### Detection of single-nucleotide polymorphisms (SNPs)

The scaffolded genome of isolate KP-5 was ordered and oriented relative to the finished genome *K. pneumoniae* PMK1 (Accession number: CP008929) via ABACAS^18^. Reads of other isolates were mapped to the rearranged KP-5 genome by CLC Genomics Workbench with default settings. Candidate SNPs were detected by the algorithm “Quality-based variant detection” of CLC Genomics Workbench. SNPs were filtered out as described previously to acquire high-quality core-genome SNPs^9^.

### Core-genome phylogenetic analysis

Fragments (≥500 bp) shared by all genomes were collected and then concatenated resulting in a pseudomolecule defined as the core genome. The detected high-quality SNPs from outbreak isolates were used for SNP-based phylogenetic reconstruction by RAxML v7.4.2^19^ with 1000 bootstrap replications under the general time-reversible model with Gamma correction (GTR+G). Assembled genomes and genomes retrieved from GenBank were aligned by ProgressiveMauve^20^. The alignment of core genomes was used for estimating the maximum likelihood (ML) phylogeny by RAxML v7.4.2 as mentioned above.

### Identification of DNA signatures and development of outbreak-specific multiplex PCR

The core genome of outbreak isolates was blasted against our local *K. pneumoniae* genome database (76 genomes with diverse sequence types). The unique fragments were extracted and blasted against GenBank (update to 20 October 2014). The non-match fragments not related to mobile genetic elements (e.g. phages, plasmids, and transposons) were considered as the DNA signatures for the outbreak clone. Primers for multiplex PCR specific to DNA signatures were designed by MPprimer^21^. All PCR-positive isolates were confirmed and further characterized by WGS.

## Results

### Outbreak description and intervention

The suspected index patient (patient 1) had been admitted to a hospital in Gambia for necrotizing pancreatitis before arriving to the Netherlands and was referred from a regional acute care hospital to the UMCG in May 2012. As an MRSA-screening test was negative before the patient arrived at the UMCG patient 1 was not placed in isolation upon admission. Subsequently, the patient was screened for ESBL-producing Enterobacteriaceae and tested positive for ESBL-KP. The patient therefore was immediately put into contact isolation which implied that medical staff was required to wear a gown and gloves when having contact with the patient. A second patient (patient 2) stayed three days at the same ward with patient 1 and was later found positive for an ESBL-KP during the ICU stay. Two further patients were identified (patient 3 and 4) staying on the same ICU as patient 2. The isolates obtained from these patients had identical antibiotic resistance features as those of patient 1 and 2. This triggered an investigation consisting of patient tracking, contact tracing and screening. Subsequently, another five patients (patient 5 to 9) sharing the same ward and facilities with patient 3 at the rehabilitation centre were found to be positive for ESBL-KP. Supplementary Fig. S1 illustrates the intra-hospital patient transfer. In November 2012, an ESBL-KP was isolated from another patient (patient 10) staying at the same ward as patients 3 and 8 who were treated in contact isolation.

In May 2013, a patient (patient 11) admitted to the neurological ICU at the UMCG was found to carry an ESBL-KP phenotypically indistinguishable from the 2012 outbreak strain on admission screening. Based on the genomic similarity between the isolate of patient 11 and the 2012 outbreak isolates, we were able to predict the grade of epidemiological relatedness (see below). Patient 11 had no contact with any of the ten positive patients. Other patients at the neurological ICU were repeatedly screened and tested negative. Patient 11 was transferred from a secondary hospital in a city about 80-kilometer south of the UMCG. Further investigation revealed that patient 11 resided in the room where patient 6, still positive for ESBL-KP, had stayed after referral from the rehabilitation centre to the secondary acute care hospital. In fact, a small outbreak with 4 patients was reported but isolates were not available for this investigation.

In September 2013, a patient (patient 12) admitted at the UMCG was identified to carry an ESBL-KP highly similar to the 2012 outbreak isolates using a clone-specific PCR (see below). No positive patients were found at the hospital during that time. Epidemiological investigation revealed that before this admission patient 12 resided in a room in November 2012 where patient 1 stayed two months before.

**Figure 1:**
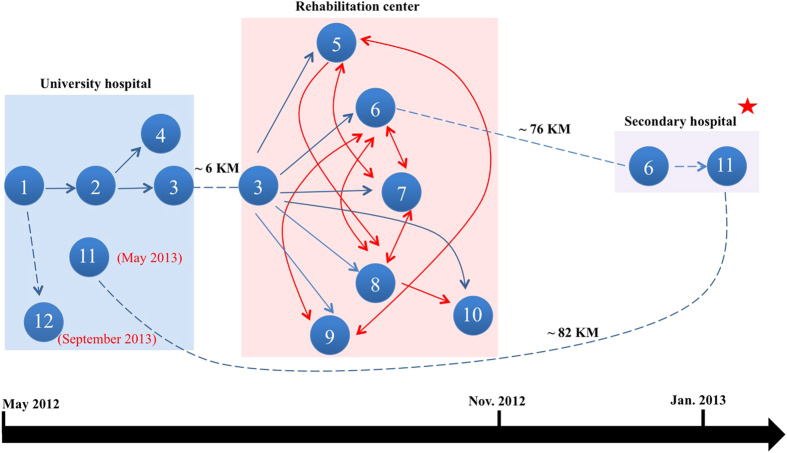
Putative transmission route of the regional outbreak. The transmission route was reconstructed by epidemiological and genomic data (see Appendix Materials and Methods for details of reconstruction methods). Each node represents a patient, and an arrow indicates a possible transmission event from one patient to another. The blue arrow with solid line represents a direct transmission event supported by both epidemiological data and genetic data, the blue arrow with dash line represents an indirect transmission (e.g. via environment) supported by epidemiological data, and the red arrow indicates the equally parsimonious transmission link which cannot be resolved by neither epidemiological data nor genetic data. The inter-institutional transfer of the patient is shown by dash line, on which the distance between institutions is indicated. The red star represents an outbreak at the secondary hospital, but the isolates were unavailable for our investigation.

In total, twelve patients positive for a phylogenetically highly related strain (see below) were identified by screening more than 120 patients from May 2012 until September 2013. Seven of them developed a clinical infection (Table 1). Figure 1 shows the most likely transmission route between the patients.

### Antimicrobial susceptibility and MLST typing

The 12 isolates (KPO-1 to KPO-12) from the 12 positive patients shared identical antibiotic resistance patterns. They were ESBL positive and resistant to amoxicillin, amoxicillin-clavulanic-acid, cefuroxime, cefotaxime, ceftazidime, gentamicin, tobramycin, co-trimoxazole, ciprofloxacin, cefepime, norfloxacin, and trimethoprim.

The MLST typing results showed that all isolates were assigned to ST15 (MLST profile: 1-1-1-1-1-1-1), which is known as the major member of the epidemic clonal group (CG) 15^3^.

### Population genomic analysis

**Figure 2:**
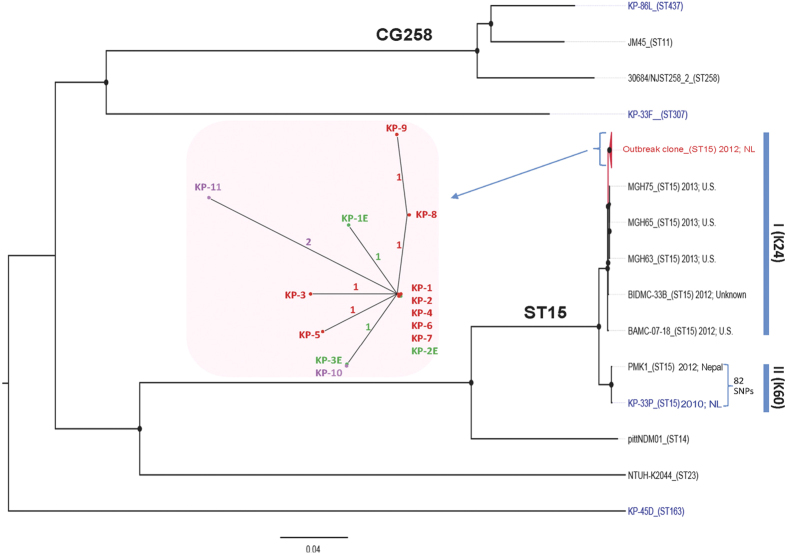
Core-genome phylogenetic analysis of *K. pneumoniae* isolates. A maximum likelihood tree was constructed based on the alignments of a 4.47 Mb genome, defined as the core genome in this study. The tree was mid-point rooted. The size of node represents the percentage of bootstrap support, and the biggest one is equal to 100. Sequence types are indicated between brackets. The isolation time and resource of all ST15 strains are shown. The cluster of outbreak isolates is simplified as a red triangle. The non-outbreak isolates sequenced in this study are in blue, and the others retrieved from GenBank are in black. The inset shows the close-up unrooted tree of outbreak isolates, in which the patient isolates are shown in red (2012) and purple (2013), and the environment isolates are in green. The number of SNPs is indicated on the branches.

Phylogenetic analysis of the core genome carried out for isolates from patients (1–4) early during the course of the investigation revealed their isogenicity. Additional isolates from patients (5–10) identified through contact tracing and screening as well as isolate KP-11 from sporadic patient 11 in 2013 all fell into the same genetic cluster (Fig. 2). The 11 isolates differed by only 7 SNPs in inter- and intragenic regions across the core genome (see Supplementary Table S1). Indeed, three isolates obtained from patients at the UMCG (KP-1, KP-2 and KP-4) and another two from patients of the rehabilitation centre (KP-6 and KP-7) were identical, i.e. they had no SNPs in the core genome. This phylogeny is concordant with available epidemiological data. We also predicted that the 2013 isolate KP-11 was derived from the outbreak clone, and differed by 2-4 SNPs from available 2012 outbreak isolates (Fig. 2).

To assess the population diversity of *K. pneumoniae* ST15, four non-outbreak ESBL-KP isolates obtained before and after the outbreak period at the UMCG (Table 1), and 10 strains retrieved from GenBank (see Supplementary Table S2 online) were included in the phylogenetic analysis. The ST15 strains were split into two distinct clades (I and II). Five ST15 strains (MGH63, MGH65, MGH75, BAMC07-18 and BIDMC-33B) isolated in US clustered with the outbreak clone (clade I) (Fig. 2). A non-outbreak ST15 strain KP-33P isolated from a patient at the UMCG in 2010 was separated from the outbreak clone, but clustered tightly with an ST15 NDM-1-producing isolate PMK1 (82 SNPs) (clade II) causing a nosocomial outbreak in Nepal 2012^22^.Another non-outbreak ST437 isolate KP-86L clustered with isolates of ST11 and ST258, belonging to the epidemic clone group 258.

### Patient screening by an outbreak-specific multiplex PCR

Three DNA signatures specific to the outbreak clone were identified. Two of them consisted of coding sequences for a hypothetical protein. The other one was located within the capsule biosynthesis region, which consisted of a glycosyltransferase-encoding gene. Primers for multiplex PCR were designed based on these three signatures (see Supplementary Table S3 online). To assess the discriminatory capability of the primers, we tested 35 isolates representing at least 20 different STs including ST15 (one isolate). The result showed that only the outbreak isolates were positive in the multiplex PCR, and that non-outbreak ST15 isolates can be successfully discriminated from the outbreak isolates (see Supplementary Fig. S2).

In September 2013, an isolate KP-12 sharing an identical antibiogram with the outbreak clone was obtained from patient 12 (Table 1), and was identified to belong to the outbreak clone as the outbreak-specific PCR was positive. In March 2015, the outbreak-specific PCR was applied to exclude the genetic relation of three isolates obtained from 3 patients staying at the same ward, and having a similar antibiogram as the outbreak isolates. Results obtained using the outbreak-specific PCR were confirmed by WGS.

### Environment screening

In total, 10 of 57 environmental samples were tested positive for ESBL-KP. To assess the genetic relatedness between environmental and patient isolates, three environmental isolates (KP-E1, KP-E2, and KP-E3) were selected for WGS (Table 1). The phylogenetic analysis showed that all three isolates clustered with the patient isolates (Fig. 2). Isolate KP-E2 (rehabilitation centre; 2012) was indistinguishable from those of five patients (KP-1, KP-2, KP-4, KP-6 and KP-7), and KP-E1 (rehabilitation centre; 2012) and KP-E3 (university hospital; 2013) differed by only up to 3 SNPs from all other patient isolates (see Supplementary Table S1 online).

### Resistome

Coding sequences associated with resistance were identified to elucidate the drug-resistance mechanisms of outbreak-associated isolates. Table 2 shows those genes detected in the outbreak isolates. All outbreak isolates shared the same resistome. The ESBL gene *bla*_CTX-M-15_ and the non-ESBL gene *bla*_SHV-28_ were detected on the plasmid and chromosome, respectively. The detection of acquired drug-resistance genes allowed us to explain the phenotypical resistance profile. Further analyses identified non-synonymous SNPs in *acrR* (A165T) and *ramR* (G56A), resulting in an amino-acid substitution in AcrR (K55N) and RamR (A19V). These proteins are regulators of AcrAB-TolC and mutations in these regulators may cause overexpression of AcrAB resulting in MDR phenotypes^23^. Analysis of three well-described mutational hotspots associated with resistance to quinolone/fluoroquinolone showed non-synonymous nucleotide changes in *gyrA* and *parC* genes, resulting in amino-acid substitutions in DNA gyrase (S83F, D87A) and topoisomerase IV (S80I)^24^.

### Virulence factors

**Figure 3:**
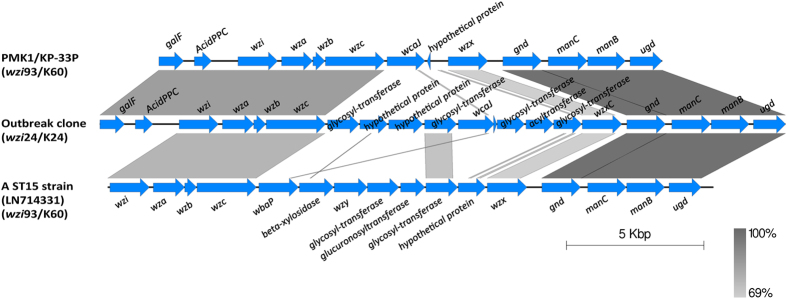
Comparison of capsular polysaccharide synthesis (*cps*) region identified from ST15 isolates. The *cps* regions of *K. pneumoniae* PMK1 and KP-33P (non-outbreak isolate of this study) were identical. The *cps* region of a ST15 strain was retrieved from GenBank. The gradients (dark to pale) of the alignment region represent the percentage of sequence identity between samples defined by BLASTn.

All outbreak isolates shared the same virulence factors. Table 3 shows the virulence factors identified in the outbreak clone, which were highly similar with those identified in the ST15 clade II isolate PMK1 from Nepal. Two hypervirulence-related iron uptake systems (ABC transporter Kfu and *Yersinia* high-pathogenicity island)^25^, were found in the outbreak clone and PMK1 but not in the non-outbreak isolate KP-33P (clade II). Although both the outbreak clone and PMK1 harboured a genomic island carrying the *Yersinia* high-pathogenicity island (encoding yersiniabactin) and a type IV(A) secretion system, the island’s compositions and its insertion site were rather different (see Supplementary Fig. S3). The BLASTn result suggests that the genomic island of the outbreak clone may have been acquired from an *Escherichia coli* stain ED 1a (Table 3). As the other ST15 clade I isolates, the capsular genotype of the outbreak clone was *wzi*24, associated with serotype K24 (http://bigsdb.web.pasteur.fr/klebsiella/klebsiella.html). This was different from that of clade II isolates PMK1 and KP-33P, which was *wzi*93, associated with serotype K60 (Fig. 3).

## Discussion

Controlling the dissemination of multidrug-resistant pathogens remains one of the major public health challenges in the 21^st^ century. Here, we share our experience in managing a regional outbreak caused by a HiRiC of ESBL-positive *K. pneumoniae* ST15. This lineage has previously been implicated in hospital outbreaks worldwide^4^,^22^,^26^,^27^. The regional and temporal dimension of this outbreak was initially unexpected but became visible when WGS data were compared with a locally available database. The outbreak involved patients from three institutions in two cities within an 80-km distance located in the same province of the Netherlands and may well have gone beyond the reach of our regional infection control capacity. The high infection rate of the outbreak clone in patients and the rebound of two patients a year after the start of the outbreak are indicators for the virulence and tenacity of this clone fulfilling the criteria of a HiRiC^28^,^29^,^30^.

The strength of WGS can be fully appreciated when considering that no obvious epidemiological association existed between a sporadic patient in 2013 (patient 11) and patients belonging to a transmission cluster one year before. A compelling degree of sequence homology consisting of 2–4 SNPs difference in the core genome between isolates from unrelated patients, led us to suggest a link, which was subsequently vindicated by epidemiological investigation. The results showed that high-resolution epidemiological typing could support infection control initiatives in unambiguously clustering patients colonized or infected with bacterial species otherwise abundant in clinical specimen.

We demonstrated that tailor-made diagnostic markers by identifying genomic signatures could highly improve the efficiency of infection control, especially for HiRiCs that may be perpetuated in health care networks. Indeed, the outbreak-specific PCR allowed us to rapidly and undoubtedly identify another “sporadic” patient (patient 12) more than one year after the start of the outbreak. Most importantly, this method enables to distinguish outbreak isolates from non-outbreak isolates even if they shared the same sequence type (i.e. ST15 isolate KP-33P). These advantages facilitate the early detection of patients, which can be greatly helpful for the prevention of clonal spread and patient treatments.

The higher resolution of WGS is also an advantage on global-scale epidemiological typing comparing to conventional methods (e.g. MLST). This greatly helps us to dissect the population structure of opportunistic bacterial pathogens by identifying HiRiCs, i.e. clonal lineages of particular public health importance. Recent work based on WGS analysis reveals that *K. pneumoniae* ST258, another HiRiC associated with the global dissemination KPC carbapenemase, is actually composed of two distinct genetic clades, which challenges conventional hypotheses on clonal descent^31^. Similarly, our study shows that the population structure of ST15 may be diverse, as two clades were formed by the ST15 isolates analysed. Full understanding of the ST15 genomic population structure will be helpful for the infection management of this HiRiC. Notably, the non-outbreak ST15 isolate KP-33P obtained in 2010 in the UMCG clustered with a ST15 NDM-1-producing Nepali outbreak isolate PMK1 (82 SNPs), suggesting that they share a common ancestor.

Noteworthy, ST15 clades I and II carried two different capsular serotypes (K24 and K60), respectively. This is similar as the recent finding that ST258 clades I and II were mainly differentiated by an ~215-kb region including *cps* genes^31^. Capsular serotypes are known to be associated with different virulence-associated traits (e.g. immune evasion and biofilm formation) in carbapenem-resistant *K. pneumoniae*^32^. The role of the different capsular serotypes in the pathogenicity of ST15 should be studied in the future to allow improved risk-assessment (i.e. identify hypervirulence- and/or epidemic-related capsule serotypes) and novel therapy (i.e. use of anti-capsule antibodies). Additionally, a *Yersinia* high-pathogenicity island encoding yersiniabactin was identified in the outbreak clone. This factor was also present in the Nepali outbreak isolate PMK1, but not in the non-outbreak ST15 isolate KP-33P. A recent study showed that yersiniabactin is essential for *K. pneumoniae* to become an effective respiratory pathogen^33^. This may be one of reasons for the high infection rate (~58.3%) observed during the outbreak and further suggests that the capacity to rapidly acquire virulence factors is important to become a HiRiC.

Analysis of the resistome identified a set of plasmid-borne resistance genes (*bla*_CTX-M-15_-*bla*_TEM-1_-*bla*_OXA-1_-*aac(6*′*)-Ib-cr*-*qnrB*1) in the outbreak clone that was found in *Enterbacteriaceae* populations spreading throughout Europe^34^,^35^,^36^,^37^,^38^. Acquisition of such resistome may confer an advantage to HiRiCs, thereby stimulating their emergence. Indeed, a highly similar resistome was identified in a new *K. pneumoniae* clone (ST1427) causing a nosocomial outbreak in our hospital^39^. Therefore, active surveillance of epidemic plasmid with certain resistome will be helpful in preventing HiRiCs dissemination.

Our study can be useful for making appropriate intervention/prevention strategies for HiRiCs in the future. First of all, the capacity to contaminate the environment facilitates the dissemination of the outbreak clone. Therefore, complete decontaminations, including cleaning and disinfection of surfaces likely to be contaminated by patients (e.g. bed and toilets) are extremely important for outbreak prevention and control. Secondly, frequent screening of patients, especially those receiving long-term healthcare and/or extensive antibiotic treatment, is required for controlling and preventing transmissions. Furthermore, extensive collaboration on diagnostics and infection prevention within a healthcare network^40^, is important for efficient infection prevention and control. For this, a shared database containing information on characteristics of circulating clones and data of intra- as well as inter-hospital patient transfer would be of great help.

In summary, our study highlights challenges in managing the outbreak of *K. pneumoniae* HiRiCs, suggesting the necessity of frequent surveillance and inter-institutional collaborations for infection/outbreak prevention and control.

## Additional Information

**How to cite this article**: Zhou, K. *et al*. Use of whole-genome sequencing to trace, control and characterize the regional expansion of extended-spectrum β-lactamase producing ST15 *Klebsiella pneumoniae*. *Sci. Rep*. **6**, 20840; doi: 10.1038/srep20840 (2016).

## Supplementary Material

Supplementary Information

## Figures and Tables

**Table 1:** Patients and *K. pneumoniae* isolates involved in this study.

Patient ID*	Location	Infection site	Sample Date	Sample ID	ST
1	University hospital	Colonization†	30/05/2012	KP-1	ST15
2	University hospital	Multiple sites#	03/06/2012	KP-2	ST15
3	Rehabilitation center	Lower respiratory tract	26/06/2012	KP-3	ST15
4	University hospital	Multiple sites#	01/07/2012	KP-4	ST15
5	Rehabilitation center	Urine tract	16/07/2012	KP-5	ST15
6	Rehabilitation center	Urine tract	14/08/2012	KP-6	ST15
7	Rehabilitation center	Urine tract	30/08/2012	KP-7	ST15
8	Rehabilitation center	Colonization†	03/09/2012	KP-8	ST15
9	Rehabilitation center	Colonization†	13/09/2012	KP-9	ST15
10	Rehabilitation center	Colonization†	24/11/2012	KP-10	ST15
11	University hospital	Lower respiratory tract	02/05/2013	KP-11	ST15
12	University hospital	Colonization†	03/09/2013	KP-12	ST15
Unrelated	University hospital	Colonization†	27/01/2012	KP-45D	ST163
Unrelated	University hospital	Blood	12/08/2010	KP-33P	ST15
Unrelated	University hospital	Blood	23/11/2012	KP-86L	ST437
Unrelated	University hospital	Blood	01/08/2013	KP-33F	ST307
Environment	Rehabilitation center	Bed	10/09/2012	KP-1E	ST15
Environment	Rehabilitation center	Toilet chair	10/09/2012	KP-2E	ST15
Environment	University hospital	Toilet chair	29/10/2013	KP-3E	ST15

^*^All isolates shared an identical antibiogram. They were ESBL positive and resistant to amoxicillin, amoxicillin-clavulanic-acid, cefuroxime, cefotaxime, ceftazidime, gentamicin, tobramycin, co-trimoxazole, ciprofloxacin, cefepime, norfloxacin, and trimethoprim and susceptible to imipenem, polymyxin B, cefoxitin and meropenem.

^#^Multiple infection sites include blood, urine tract, lower respiratory tract, and central venous line.

^†^Colonization was defined by positive cultures acquired from throat and/or rectum samples.

**Table 2:** Coding sequences related to drug resistance present in the outbreak clone.

Category	Genetic context	Resistance phenotype	
Drug-resistance gene			Best Hit*(Identity)
*bla*_TEM-1_	Plasmid	Penicillins, narrow-spectrum cephalosporins, inhibitor-sensitive	100% (JF910132)
*bla*_OXA-1_	Plasmid	Penicillins, inhibitor-resistant	100% (J02967)
*bla*_SHV-28_	Chromosome	Penicillins, narrow-spectrum cephalosporins, inhibitor-sensitive	100% (AF538324)
*bla*_CTX-M-15_	Plasmid	Penicillins, extended-spectrum cephalosporins, aztreonam	100% (DQ302097)
*strAB*	Plasmid	Streptomycin	100% (AF321551, M96392)
*aac(6’)-Ib-cr*	Plasmid	Aminoglycosides, fluoroquinolones	100% (DQ303918)
*aac(3)-II*	Plasmid	Gentamicin, tobramycin, netilmicin, sisomicin	100% (JX424423)
*qnrB1*	Plasmid	Quinolones, fluoroquinolones	100% (NG_036203.1)
*sul2*	Plasmid	Sulfonamides	100% (GQ421466)
*dfrA14*	Plasmid	Trimethoprim	100% (GU726917)
*fosA*	Chromosome	Fosfomycin	99% (CP009114)
*tetA*(A)	Plasmid	Tetracyclines	100% (AJ517790)
Efflux pump (familiy)			
*acrAB-tolC* (RND)†	Chromosome	Aminoglycosides, beta-lactams, tigecycline, macrolides	PMK1 (100%)
*acrD* (RND)	Chromosome	Aminoglycosides, deoxycholate, fusidic acid, novobiocin	PMK1 (100%)
*mdtABC* (RND)	Chromosome	Deoxycholate, novobiocin, bile salt	PMK1 (100%)
*oqxAB* (RND)	Chromosome	Chloramphenicol, fluoroquinolones, trimethoprim	99%/100% (CP009461)
*bcr* (MF)	Chromosome	Bicyclomycin, sulfathiazole	PMK1 (100%)
*emrAB* (MF)	Chromosome	Nalidixic acid, hydrophobic compounds	PMK1 (100%)
*fsr* (MF)	Chromosome	Fosmidomycin	PMK1 (100%)
*mdfA* (*kdeA*) (MF)	Chromosome	Aminoglycosides, fluoroquinolones, chloramphenicol	PMK1 (100%)
*mdtG* (MF)	Chromosome	Deoxycholate, fosfomycin	PMK1 (100%)
*mdtH* (MF)	Chromosome	Enoxacin, norfloxacin	PMK1 (100%)
*mdtL* (MF)	Chromosome	Chloramphenicol	PMK1 (100%)
*smvA*(MF)	Chromosome	Acriflavine, quaternary ammonium compounds	PMK1 (100%)
*sugE* (SMR)	Chromosome	Benzalkonium chloride, ethidium bromide	PMK1 (100%)
*mdtK* (MATE)	Chromosome	Acriflavine, norfloxacin	PMK1 (100%)
*macAB-tolC* (ABC)	Chromosome	Macrolides	PMK1 (100%)
Gene with point mutation	Amino-acid change		
*gyrA*	S83F, D87A	Quinolones, fluoroquinolones	
*parC*	S80I	Quinolones, fluoroquinolones	

^*^The best hit is defined by Blastn on http://blast.ncbi.nlm.nih.gov/Blast.cgi. All matches are *K. pneumoniae* with 100% coverages.

^†^There was a nonsynonymous SNP causing an amino-acid substitution (K55N) in AcrR.

**Table 3:** Coding sequences related to virulence factors present in the outbreak clone.

Virulence factor	Gene(s) detected	Best hit*(coverage, identity)	Genetic context
Adhesin			
Type 1 fimbriae	*fimBEAICDFGHK*	PMK1 (100%, 99%)	Chromosome
Type 3 fimbriae	*mrkABCDF*	PMK1 (100%, 100%)	Chromosome
Kpa fimbriae	*kpaABCDE*	PMK1 (100%, 100%)	Chromosome
Kpb-like fimbriae^±^	*kpbRABCD*_*2*_	PMK1 (100%, 100%)	Chromosome
Kpe fimbriae#	*ΔkpeA, kpeBCD*	PMK1 (100%, 100%)	Chromosome
Kpf fimbriae	*kpfRABCD*	PMK1 (100%, 100%)	Chromosome
Kpg fimbriae	*kpgABCD*	pittNDM01 (100%, 99%)	Chromosome
Fim2ǂ	*fim2AIC*, *fim2D*::*tnpA*, *fimF-K*	PMK1 (100%, 100%)	Chromosome
ECP (Mat) fimbriae	*ecpRABCDE*	PMK1 (100%, 100%)	Chromosome
polysaccharide adhesin	*pgaABCD*	PMK1 (100%, 100%)	Chromosome
Capsule			
*wzi*24/K24	*galF-wzc, wcaJ-ugd*^†^ (Fig. 3)	ATCC 43816 KPPR1 (50%, 95%)	Chromosome
Iron uptake system			
Fep-ent (Enterobactin)	*fepA-entD, fes-entF, fepDGC, ybdA, fepB, entCEBA*	PMK1 (100%, 100%)	Chromosome
Iuc (Aerobactin)	*iutA*	PMK1 (100%, 100%)	Chromosome
IroA (Salmochelin)	*iroN*	PMK1 (100%, 100%)	Chromosome
Fhu (Ferrichrome)	*fhuACDB*	PMK1 (100%, 100%)	Chromosome
ABC transporter Sit (Ferrous iron)	*sitABCD*	PMK1 (100%, 100%)	Chromosome
ABC transporter Eit (Iron/B12/siderophore/hemin)	*eitABCD*	PMK1 (100%, 100%)	Chromosome
ABC transporter Kfu (Ferric iron)	*kfuABC*	PMK1 (100%, 100%)	Chromosome
High-pathogenicity island (Yersiniabactin)	*ybtPQXS, ybtA-irp2-irp1-ybtUTE-fyuA*	*E. coli* ED 1a (100%, 99%)	Chromosome
Feo (Ferrous iron)	*feoABC*	PMK1 (100%, 100%)	Chromosome
Hmu (Hemin/hemoprotein)	*hmuRSTUV*	PMK1 (100%, 100%)	Chromosome
Nitrogen source utilization			
Urease	*ureDCBAEFG*	PMK1 (100%, 100%)	Chromosome
Secretion system			
F-type T4SS	*traALEKBVCGFHNUW, trbC*†	JM45 plasmid p1 (100%, 99%)	Plasmid
T4SS (type IVA)	*virB1-2*, *virB4-6*, *virB8-11*, *virD4*	*E. coli* ED 1a (100%, 99%)	Chromosome
T6SS	*impH-G, imcF, vgrG, ompA, impJ, clpV*†	PMK1 (100%, 100%)	Chromosome

^*^The best hit is defined by Blastn on http://blast.ncbi.nlm.nih.gov/Blast.cgi. The species name is not shown if the matches are from *K. pneumoniae*. ^±^The adhesin gene *kpbD* was replaced by a different adhesin gene *kpdD*_*2*_ (the gene was named in this study).

^#^The subunit gene *kpeA* was replaced by a gene encoding Tn903 transposase.

^ǂ^The intact gene cluster of this fimbriae was identified on a genomic island KpGI-5 (JN181158) of *K. pneumoniae* KR116.

^†^Only conserved/hallmark genes are shown here.
