# Supplementary material for: Use of whole-genome sequencing to trace, control and characterize the regional expansion of extended-spectrum β-lactamase producing ST15 Klebsiella pneumoniae
Source: Sci Rep. 2016 Feb 11;6:20840. doi: 10.1038/srep20840 (PMC4749987; doi:10.1038/srep20840)
Supplement: Supplementary Information [file srep20840-s1.doc]

**Use of whole-genome sequencing to trace, control and characterize the regional expansion of extended-spectrum β-lactamase producing ST15 *Klebsiella pneumoniae***

**Kai Zhou^1, 3^, Mariette Lokate^1^, Ruud H. Deurenberg^1^, Marga Tepper^2^, Jan P. Arends^1^, Erwin GC Raangs^1^, Jerome Lo-Ten-Foe^1^, Hajo Grundmann^1^, John W.A. Rossen^1#^, Alexander W. Friedrich^1^*^#^**

**^1^**Department of Medical Microbiology, University of Groningen, University Medical Center Groningen, Groningen, Netherlands; ^2^Department of Rehabilitation Medicine, Center for Rehabilitation, University of Groningen, University Medical Center Groningen, Netherlands; ^3^State Key Laboratory for Diagnosis and Treatment of Infectious Diseases, Collaborative Innovation Center for Diagnosis and Treatment of Infectious Diseases, the First Affiliated Hospital of Medicine School, Zhejiang University

**Supplementary Table**

**Supplementary** **Table S1.** **SNPs detected among outbreak isolates.**

| **SNP** | **KP-1** | **KP-2** | **KP-3** | **KP-4** | **KP-5** | **KP-6** | **KP-7** | **KP-8** | **KP-9** | **KP-10** | **KP-11** | **KP-1E** | **KP-2E** | **KP-3E** | **Coding region change** | **Amino acid change** |
| --- | --- | --- | --- | --- | --- | --- | --- | --- | --- | --- | --- | --- | --- | --- | --- | --- |
| **1** | . | . | . | . | A->C | . | . | . | . | . | . | . | . | . |  |  |
| **2** | . | . | . | . | . | . | . | A->C | A->C | . | . | . | . | . | Proline dehydrogenase (Proline oxidase) (EC 1.5.99.8):488T>G | Val163Gly |
| **3** | . | . | C->T | . | . | . | . | . | . | . | . | . | . | . | Ribose operon repressor:766C>T | Gln256* |
| **4** | . | . | . | . | . | . | . | . | T->G | . | . | . | . | . |  |  |
| **5** | . | . | . | . | . | . | . | . | . | . | T->C | . | . | . | Putrescine transport ATP-binding protein PotG (TC 3.A.1.11.2):152T>C | Leu51Pro |
| **6** | . | . | . | . | . | . | . | . | . | . | C->G | . | . | . | Putative esterase:102G>C |  |
| **7** | . | . | . | . | . | . | . | . | . | . | . | G->T | . | . | Phosphonate ABC transporter ATP-binding protein (TC 3.A.1.9.1):569C>A | Ala190Glu |
| **8** | . | . | . | . | . | . | . | . | . | G->A | . | . | . | G->A | Phosphonate ABC transporter ATP-binding protein (TC 3.A.1.9.1):229C>T | Gln77* |

Note: SNPs are indicated in red and loci without mutations are shown as dots.

**Supplementary Table S2. *K. pneumoniae* strains retrieved from GenBank.**

| **Isolate** | **ST** | **Accession number** |
| --- | --- | --- |
| JM45 | ST11 | CP006659 |
| pittNDM01 | ST14 | CP006798 |
| PMK1 | ST15 | CP008929 |
| MGH63 | ST15 | NZ_JJNB00000000 |
| MGH65 | ST15 | NZ_JMZA00000000 |
| MGH75 | ST15 | NZ_JJNK00000000 |
| BAMC07-18 | ST15 | NZ_JRQE00000000 |
| BIDMC-33B | ST15 | NZ_JNVK00000000 |
| NTUH-K2044 | ST23 | NC_012731 |
| 30684/NJST258_2 | ST258 | CP006918 |

**Supplementary Table S3. Primers used for clone-specific multiplex PCR.**

|  | **Fw (5'-3')** | **Rev (5'-3')** | **Product size** |
| --- | --- | --- | --- |
| **Pair 1** | AACGTGTCTGGCGCTGTTGAGC | TCGACCTCGTAGCCTATTGCTGC | 122 bp |
| **Pair 2** | TCAACCGGAACATACCCAAGGACA | TCAAGCAAGGCGAAAACACTCAGTT | 220 bp |
| **Pair 3** | TCGCGGCTTTGAATTCAGGCGT | CGCCAAATTCGTTGGTGGCTGC | 422 bp |

**Supplementary Figure**

**Supplementary Fig. S1 Movements of positive patients before and during the 2012 outbreak.** The white spots with isolate names shown on the bar represent the first ESBL-KP isolate obtained from each patient which was sequenced in this study. Patient 9 (P-9) was sampled at home after discharging and was identified by contact tracing. Different wards are indicated by different colors. Ward C belongs to the rehabilitation centre. Ward D is the ICU of the university hospital.

**Supplementary Fig. S2 Validation of the outbreak-specific multiplex PCR.** Lane M: DNA ladder; Lane 7-11: outbreak isolates; Lane 1-6 and 13-17: non-outbreak isolates (Lane 6 is a ST15 non-outbreak isolate); Lane 12: positive control; Lane 18: negative control.

**Supplementary Fig. S3 Comparison of the genomic island carrying a high-pathogenicity island encoding yersiniabactin and a T4SS.** The sequence types of isolates included here are shown between brackets. The tRNA was shown as a brown bar, and the insertion site of the genomic island was indicated by the arrow. The gradients of the alignment region represent the percentage of sequence identity between samples defined by BLASTn. The blue and red indicate direct or inverted conserved segments, respectively.

**Supplementary Materials and Methods**

*Nucleotide sequence accession numbers*

The Whole Genome Shotgun BioProject for our sequenced *K. pneumoniae* isolates has been deposited at DDBJ/EMBL/GenBank under the accession of JXIU00000000 (KP-1), JXIV00000000 (KP-2), JUBN00000000 (KP-3), JXIW00000000 (KP-4), JUBO00000000 (KP-5), JXIX00000000 (KP-6), JXIY00000000 (KP-7), JXIZ00000000 (KP-8), JXJA00000000 (KP-9), LDOY00000000 (KP-10), JXJB00000000 (KP-11), JXJC00000000 (KP-1E), JXJD00000000 (KP-2E), JXJE00000000 (KP-3E), JXJF00000000 (KP-45D), JXJG00000000 (KP-33P), JXJI00000000 (KP-86L) and JXJJ00000000 (KP-33F), respectively.

*Reconstruction of the transmission chain*

Either epidemiological or genomic data were used for tracing the patient-to-patient transmission events. Using the epidemiological data, a transmission event was predicted if the presence of two patients overlapped in the same ward at the same time before cultures of the potential recipient were found positive^9^. An R package Outbreaker^41^ was used to predict the transmission chain by using genomic data (SNPs) and sampling dates. Since transmissions occurred first in the hospital and later in a rehabilitation centre, two respective computations were performed on the samples according to their isolation location with exception of KP-3, which was shared by both computations. The threshold of posterior support of ancestry was set at 0.5.

*Multiplex PCR*

PCR amplification was performed by 5 min at 95˚C, 35 cycles of 30s at 95 ˚C, 30s at 59 ˚C, and 60s at 72 ˚C, followed by 5min at 72 ˚C. The reaction volume is 20µl.

**References:**

41. Jombart, T. et al. Bayesian reconstruction of disease outbreaks by combining epidemiologic and genomic data. PLoS Comput. Biol. 10, e1003457, doi: 10.1371/journal.pcbi.1003457 (2014).
